# Comparative genome analysis of a large Dutch *Legionella pneumophila *strain collection identifies five markers highly correlated with clinical strains

**DOI:** 10.1186/1471-2164-11-433

**Published:** 2010-07-15

**Authors:** Ed Yzerman, Jeroen W den Boer, Martien Caspers, Arpit Almal, Bill Worzel, Walter van der Meer, Roy Montijn, Frank Schuren

**Affiliations:** 1 Regional Public Health Laboratory of Haarlem, Haarlem, the Netherlands; 2 Department of Microbiology, TNO, Zeist, the Netherlands; 3 Genetics Squared, Ann Arbor, MI 48108, USA; 4 Vitens Research, Leeuwarden, the Netherlands

## Abstract

**Background:**

Discrimination between clinical and environmental strains within many bacterial species is currently underexplored. Genomic analyses have clearly shown the enormous variability in genome composition between different strains of a bacterial species. In this study we have used *Legionella pneumophila*, the causative agent of Legionnaire's disease, to search for genomic markers related to pathogenicity. During a large surveillance study in The Netherlands well-characterized patient-derived strains and environmental strains were collected. We have used a mixed-genome microarray to perform comparative-genome analysis of 257 strains from this collection.

**Results:**

Microarray analysis indicated that 480 DNA markers (out of in total 3360 markers) showed clear variation in presence between individual strains and these were therefore selected for further analysis. Unsupervised statistical analysis of these markers showed the enormous genomic variation within the species but did not show any correlation with a pathogenic phenotype. We therefore used supervised statistical analysis to identify discriminating markers. Genetic programming was used both to identify predictive markers and to define their interrelationships. A model consisting of five markers was developed that together correctly predicted 100% of the clinical strains and 69% of the environmental strains.

**Conclusions:**

A novel approach for identifying predictive markers enabling discrimination between clinical and environmental isolates of *L. pneumophila *is presented. Out of over 3000 possible markers, five were selected that together enabled correct prediction of all the clinical strains included in this study. This novel approach for identifying predictive markers can be applied to all bacterial species, allowing for better discrimination between strains well equipped to cause human disease and relatively harmless strains.

## Background

Identifying the genetic factors that influence the pathogenic potential of microorganisms is of the greatest importance in trying to gain better control of infectious diseases. *Legionella pneumophila*, the causative micro-organism of Legionnaires' disease (LD), is an aquatic bacterium that can be found in numerous water sources. Several aerosol-producing systems have become associated with LD outbreaks (including cooling towers, saunas, and whirlpool spas). The financial, economic and social impacts of these outbreaks can be enormous and many countries have implemented governmental laws or guidelines to prevent the growth of *Legionella *bacteria in potential sources. In most regulations, disinfection strategies are used independent of the isolated *Legionella *strains, as there is no reliable method for differentiating between clinical and environmental strains. In The Netherlands, this has led to a confounding situation in which, despite an estimated expenditure of several billion euros since 1999 (when 32 people died in a single outbreak of LD; [1]) the incidence of LD has steadily increased [2]. One explanation for this might be the lack of focus in combating *Legionella *risks: all *Legionella *species are considered equally dangerous, with no attention being given to clinical data on LD, even though over 95% of LD cases are caused by *L. pneumophila*. Furthermore, within the species *L. pneumophila*, serogroup 1 is involved in over 80% of cases [3]. As has already been demonstrated with guinea pig models in the 1980s, some serogroup 1 subtypes are more virulent than others [4]. This difference in virulence across serogroups and subtypes appears to be due mainly to the better-developed replicating and apoptosis skills of *L. pneumophila *strains, especially in serogroup 1 [5]. Epidemiological studies have also provided some clues on virulence. A particular lipopolysaccharide epitope, recognized as MAb 2 by typing with a panel of monoclonal antibodies obtained from a range of geographical locations, is more frequently expressed on patient derived isolates than on environmental isolates of serogroup 1 strains. Furthermore, fingerprinting of *L. pneumophila *strains has shown that the genotypes of isolates from patients and from the environment differ markedly [6] indicating that a genetic base for differences in virulence does exist. However, the nature of this genetic base has been fully unclear until now. Also genomic analysis of 217 *L. pneumophila *strains collected world-wide could not identify specific hybridization profiles differentiating clinical and environmental strains [7]. To identify molecular markers related to virulent behavior, we have combined two different worlds. We used comparative genome hybridization (CGH) based on a mixed-strain microarray containing genetic information from both clinical and environmental isolates. This molecular analysis was performed with a large collection of well-described bacterial isolates derived from a large surveillance study in The Netherlands in which both patient-derived strains and environmental strains were collected. We investigated whether the application of such a molecular analysis on a well-defined strain collection was capable of identifying DNA markers that would allow for discrimination between clinical and environmental isolates. In this way we have identified five markers that together constitute a model highly predictive for clinical strains.

## Results

### Strain collection

**Figure 1:**
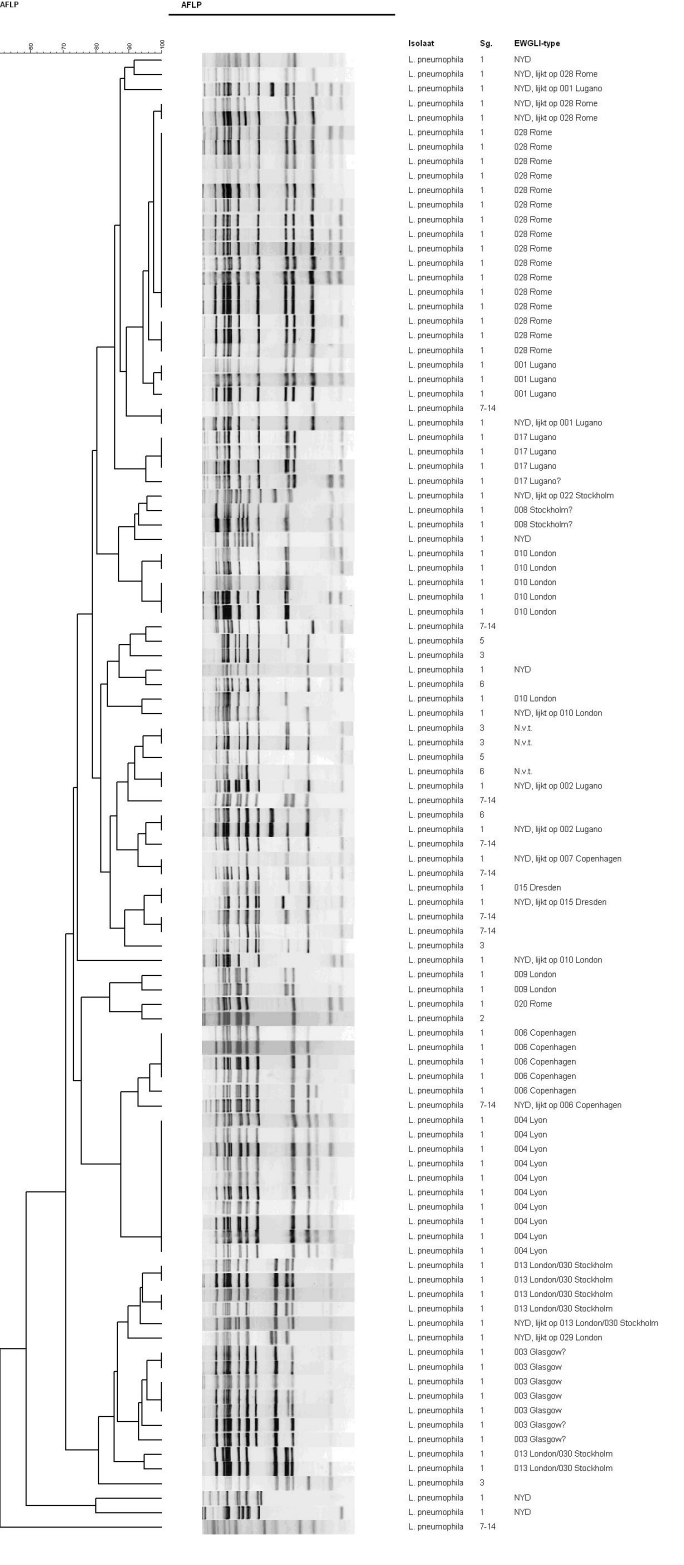
**AFLP analysis of *L. pneumophila *serogroup 1 isolates**. AFLP analysis shows genomic diversity between a representative subset of 102 *L. pneumophila *strains used for further analysis in this study. For each strain serogroup type (Sg.) and assigned EWGLI-type are shown. The variety of patterns and presence of several different EWGLI-types indicates the random selection of strains representing the natural genomic diversity within this species.

The basis of this study is formed by the strain collection from the Dutch Legionella surveillance program. This collection encompasses all patient-derived strains from notified cases in The Netherlands in the period from 2002-2006 and all environmental strains that were collected in an attempt to identify the source of infection for those patients. The *L. pneumophila *isolates from the patients' environments were genotypically compared with the patient strain. Non-matching isolates from the environment were defined as environmental. Patient strains and matching isolates from the environment were defined as clinical. The collection of environmental strains was completed with isolates from sources located in surroundings geographically free of LD patients according to the registration history of notified cases. All *L. pneumophila *strains were serotyped, and the serogroup 1 isolates were analyzed by Amplified Fragment Length Polymorphism (AFLP) analysis according to the European Working Group on Legionella Infections (EWGLI) format [8]. In total, 257 strains from this collection of clinical and environmental *L. pneumophila *isolates were selected for further analysis in this study (see additional file 1). A representative part of this selection (102 isolates) was analyzed by AFLP (Figure 1). A broad variety of patterns is present, indicating the genomic diversity within this species and the random selection of the isolates from the natural genomic diversity of *L. pneumophila *strains.

### Microarray construction and unsupervised analysis

**Figure 2:**
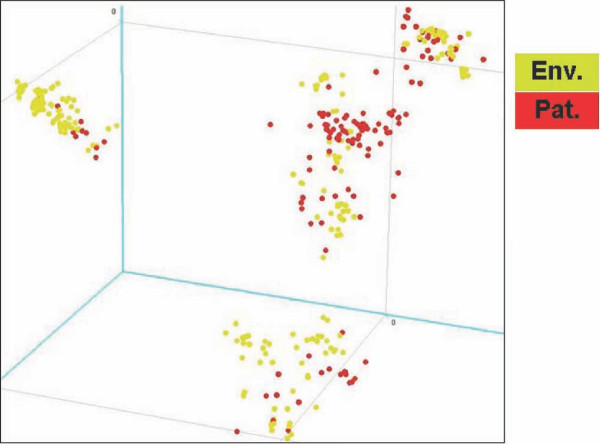
**Genomic diversity based on Principal Component Analysis (PCA)**. All 346 microarray data sets (linearly distributed values used, based on the selection of 480 markers) were subjected to Principal Component Analysis to determine whether this would lead to subgrouping of patient-derived or environmental strains of *L. pneumophila*. Each data set is represented as a single dot in an n-dimensional space of which the three main components (covering most of the overall variation covered within these data) are shown. It is clear from this analysis that no clear differences between environmental and patient-derived strains are detected based on overall genome composition.

Comparative genome hybridization experiments were performed with a mixed-genome *L. pneumophila *microarray. For the construction of this microarray, eight *L. pneumophila *strains were selected based on their diversity (both patient-derived and environmental strains were used; for details see additional file 1). This microarray consisting of 3360 genomic fragments was used to analyze the genome composition of the collection of 257 unique *L. pneumophila *strains by comparing labeled DNA from each strain with a reference containing labeled DNA from the mixture of strains used for array construction. In total, 346 datasets were generated each representing a fingerprint encompassing over 3000 different markers. The data for all spots on all microarrays were calculated as ratios between the tester strain and the reference with normalization for experimental differences (for details see methods section). These data were used for unsupervised statistical analysis of the relationships between all strains with respect to genomic composition. Principal component analysis of all 346 CGH-data sets resulted in a good overview and allowed us to draw a number of conclusions important for follow-up work. First, results from repeat experiments (89 in total, representing both technical and biological replicates) showed the high reproducibility of this approach (data not shown). Next, this analysis also showed that the overall genomic patterns (consisting of over 3000 markers per strain) of the environmental *L. pneumophila *isolates were found throughout the distribution of the patterns obtained from the patient-derived strains (Figure 2), indicating that no genome wide differences between the two groups exist. Furthermore, no small outlier populations or single outlier strains were observed with the exception of a small number of datasets obtained from *Legionella *species other than *L. pneumophila. *These strains were not present in the 257 strain dataset and were excluded from all further analyses. This result indicated to us that the genomic diversity of the species was well covered by the 257 selected strains and that there was no need for analyzing additional strains.

### Marker reduction and binarization

Unsupervised statistical analysis of the data obtained for the 257 strains did not allow for discrimination between clinical and environmental strains. Since these analyses are based on overall trends in data this inability to discriminate is not surprising. It is much more likely that a small part of the genome is involved in a specific genetic trait such as pathogenic potential. Therefore, supervised multivariate statistical analysis of the complete data set was also used in order to find support for the hypothesis that discrimination between clinical and environmental strains is feasible. Although this analysis indicated that discrimination was possible the method used for this analysis (partial least square discriminant analysis) was not optimally suited for reliable statistical significance analysis, nor for selecting a minimal set of predictive markers. Therefore, a different data analysis approach was taken. First, the number of markers was reduced based on the observation that approximately 80% of the 3,000 markers were present in all strains and therefore encompassed the *L. pneumophila *core genome. The remaining 20% of the markers showed clear variation in presence between individual strains and encompassed the variable part of the genome. Further reduction of the number of markers was achieved by taking only a few representatives in those cases where multiple markers showed nearly identical patterns over the complete data set (strongly suggesting partial overlap or close linkage in the genome). In total, 480 markers were selected in this way and this selection was used for all further analyses (additional files 2 and 3).

**Figure 3:**
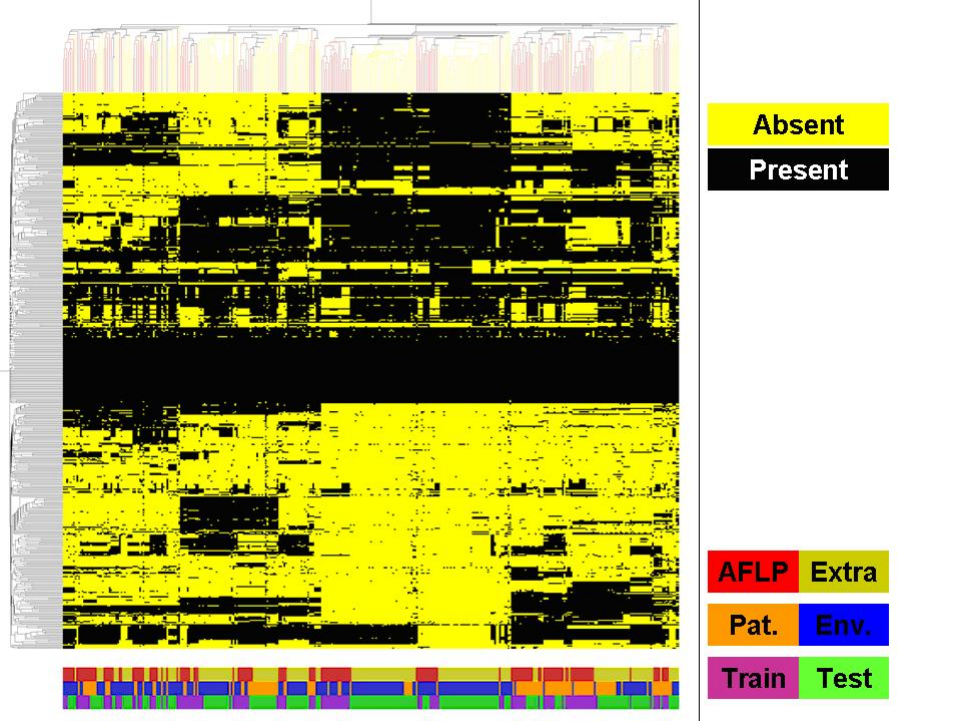
**Genomic diversity based on hierarchical clustering analysis**. Hierarchical clustering analysis was performed on all 346 genomic datasets generated in this study. Data were clustered both in columns (representing datasets) and rows (representing 480 spots on the array) with Pearson correlation distances and average linkage. Binarized data were used for this analysis. Yellow represents absence of signal, black represents presence of signal. The color-coded bars on the bottom show the distribution of the strains used for AFLP analysis as shown in Figure 1 (red) and the additional strains (yellow-green), the distribution of patient (orange) and environmental (blue) strains and the distribution of training (purple) and test (green) set strains. In all cases the random distribution of the groups is clearly visible.

Next to reducing the number of markers another preprocessing step was included. Closer examination of the selected markers in most cases suggested a binary distribution of values, most likely representing either the apparent presence or absence (or presence but with a low sequence homology preventing hybridization) of specific genetic elements. Since this binary distribution probably represents the real-world situation among strains and also favors the practical applicability of analyzing selected markers, we decided to continue our analyses by converting our linearly distributed data into binary values representing the presence or absence of each marker. A number of methods have been described in recent years to perform binarization of markers, but none of these performed sufficiently well. Some of these methods are rather simple, being based on a preset cut-off (*e.g. *0.5) or a standard deviation-based classification. We tried the more advanced GACK approach [9] based on the shape of signal ratio distributions; although this method worked reasonably well in general, we were not satisfied by its overall performance. One possible explanation for this is the fact that this method was developed for single strain (genome) microarrays whereas the microarray used in this study is based on multiple genomes. Another explanation is the fact that the generalization of cut-off selection, which is also part of this method, leads to unsatisfactory results for part of the data. We therefore had a closer look at our data and found an alternative solution which is based on the distribution of all ratios for each individual marker to determine an individual marker-dependent cut-off value. In cases where no clear cut-off could be determined all data points were classified as being present. In cases where multiple cut-offs were possible we decided to apply multiple cut-offs by splitting up these markers and using these multiple variants of the same marker for further data analysis. Other available binarization methods tend to ignore this last category in particular. Some examples of this binarization approach are shown in additional file 4. Binarized data are given in additional files 5 and 6. Hierarchical clustering of the binarized data shows the enormous amount of variation still present in this data set (Figure 3). A number of conclusions can be drawn from this analysis. First, although some clustering can be observed for the strains for which AFLP data were generated this clustering does not indicate segregation into specific groups (Figure 3). Next, the absence of clear discrimination between environmental strains and patient-derived strains is visualized (Figure 3). Finally, the distribution of strains used as training and test set (described in more detail in the next section) shows a fair amount of random variation (Figure 3). No single markers were detected which enabled clear discrimination between patient-derived and environmental strains based on the overall datasets. Since no clear discrimination between environmental and patient-derived strains was obtained with these unsupervised analyses attention was shifted towards the application of supervised analyses. The binary data were used as input data for these analyses.

### Selection of discriminating markers

Many supervised statistical analysis methods have been described for analyzing microarray data [10]. A limitation of many of these methods is the fact that they use a data reduction approach which makes it difficult to select a minimal set of markers. We have chosen as a method for selecting a minimal set of predictive markers a machine learning technique called Genetic Programming [11-13]. Genetic Programming (GP) is a machine learning approach that provides a collection of solutions to biodiagnostic problems by creating classifier programs using a subset of the available input. A "genetic pool" of candidate classification programs was created by randomly choosing inputs and arithmetic and Boolean operators that work with the inputs selected, and evolving successive generations of programs were through selection and recombination. The accuracy of a program in correctly classifying the samples according to pre-specified classes is used to calculate a fitness measure for each program. Fitness was determined by calculating the area under the curve (AUC) for the receiver operating characteristic (ROC) of a program generated by the GP system [14]. Evolution was driven by improving the AUC (fitness score) so as to yield rules with high sensitivity and specificity. The complexity of the rules generated was also restricted to prevent overfitting of the training data. The programs were evolved over many generations resulting in progressively better and more accurate programs. The stopping criterion was either a perfect classifier within the limits of program size and complexity, or the generation limit was reached. Table 1 shows a list of the inputs, operators, and GP parameters such as fitness function, population size, crossover and mutation frequencies, etc.

**Table 1:** GP control parameters

Elitism	True
Inputs	LePn.011A2-b, LePn.019H4, LePn.010B12, LePn.008D6, LePn.024B1

Operators	=, >, <, > =, < =, and, not, or, ?, nand, xor, nor

Fitness Function	AUC

Population Size	10000

Cross Over Rate	0.7

Mutation Rate	0.3

Generation Limit	100

The genetic programming system used is a patented system [15] and adapted for use in bio-classifier problems such as that described here. The source code for this implementation has not been published, but other than the representation of programs, it is a fairly standard implementation of steady-state genetic programming using both crossover and mutational operators on tree-like structures. This system was adapted to evolve classifier programs by creating mathematical models and assigning a slice point based on maximizing the AUC for the model [16].

Reducing the dimensionality of the data is of foremost importance in most analysis programs. Here we have used gene frequency (*i.e. *the count of 'gene occurrences' in the best performing rules), to identify the markers that provide the most value for developing an accurate classifier, an approach that borrows from the "symbolic discriminant analysis" method [17]. Cross-validation-based resampling [18] was used to estimate the ability of the classifier to generalize to unseen samples, giving an approximation of its robustness. Classifier sets (ensembles) are composed from the best rules from each fold. The ensemble is then "polled" with each rule in the ensemble voting on whether a sample belongs to a target class. If the majority of the rules (in this case four or more) agree that the sample belongs to the target class (in this case, clinical), the ensemble predicts the sample belongs to the target class. Aggregate performance of these ensembles on the test folds was taken as the predictor of the classification error, and the selected ensemble was the one with the smallest test error.

The approach used for the *Legionella *strain data was as follows:

First, the data set was split in two: a learning set of 133 samples was used for creating a predictive model and a reserved validation set consisting of 213 samples was kept apart for testing of the model. Using the 133 samples (representing 109 unique strains) from the learning set, which was classified as approximately 50% clinical and 50% environmental, a predictive rule was developed that identified clinical strains. The goal was to identify 100% of the clinical strains correctly while keeping the number of misclassified environmental strains as low as possible (*i.e.*, maximizing sensitivity while minimizing the loss of specificity). To do this, the control parameters of the genetic programming system were modified to give a larger bias towards achieving higher sensitivity compared to the specificity. A sevenfold cross-validation-based resampling of the 133 samples was used, as this provided a large enough training set while leaving a reasonable test set in each fold [14,18]. From the results of the first set of analytical runs, the 5 most frequently used markers were selected based on the frequency of the genes appearing in the best rules of 265 runs using the average length of the rules created in each run. The genetic programming system was then run again with only these 5 markers to develop an ensemble of classifiers that show good performance [17]. This analysis resulted in the selection of an optimal model out of the various solutions on offer; this model consisted of 5 markers and resulted in a sensitivity of 100% and a specificity of 62% (Table S6). The 5 marker model consists of 7 rules containing all relevant interrelationships between the 5 selected markers. The majority vote of these 7 rules determines the final prediction (Table 2).

**Table 2:** The 7 rules of the predictive model describing all relevant relationships between the 5 selected markers

Rule No.	Rule
1	If (LePn.011A2-b < LePn.019H4) then [not(LePn.011A2-b = LePn.010B12)] else [(LePn.011A2-b = LePn.024B1) and (LePn.008D6 = LePn.010B12)]

2	If (LePn.011A2-b < LePn.010B12) then [not(LePn.019H4 < LePn.024B1)] else [(LePn.008D6 > LePn.011A2-b) nor (LePn.008D6 < LePn.024B1)]

3	If (LePn.011A2-b < LePn.010B12) then [not(LePn.024B1 > LePn.019H4)] else [(LePn.010B12 < LePn.008D6) nor (LePn.024B1 > LePn.008D6)]

4	If (LePn.019H4 = LePn.008D6) then [(LePn.024B1 < = LePn.019H4)] else [(LePn.019H4 < LePn.024B1) nor (LePn.010B12 = LePn.011A2-b)]

5	If (LePn.019H4 = LePn.008D6) then [(LePn.008D6 > = LePn.024B1)] else [(LePn.010B12 < = LePn.011A2-b) nor (not (LePn.024B1 < = LePn.019H4))]

6	If (LePn.019H4 = LePn.008D6) then [not(LePn.024B1 > LePn.019H4)] else [(LePn.024B1 > LePn.019H4) nor (LePn.011A2-b > = LePn.010B12

7	If (LePn.019H4 = LePn.008D6) then [not(LePn.019H4 < LePn.024B1)] else [(LePn.010B12 = LePn.011A2-b) nor (LePn.019H4 < LePn.024B1)]

The 7 rules of the predictive model describing all relevant relationships between the 5 selected markers. Each rule "votes" for a sample as being a clinical sample if the result of the rule evaluates to 'True' when the Boolean value for each probe is entered. If 4 or more rules in the table agree that a given sample is clinical, then the "meta-rule" of these 7 rules predicts that the sample is clinical.

### Validation of the model

Since the model obtained was based on a learning set of 133 data sets (representing 109 unique strains) we were able to use an additional 213 data sets (representing 148 unique strains) not employed for building the model as a reserved validation set. Application of the model to these additional data sets confirmed the results from the learning set: a sensitivity of 100% and a specificity of 69% were obtained with this validation set (Table 3, additional file 7). Although this is not an external validation in a formal sense (the samples were partly taken from the same Dutch population, the raw data were generated by the same laboratory, and the same people processed the raw data to binary values and analyzed all data) these results clearly support the validity of the predictive model.

**Table 3:** Prediction results when testing the data obtained on a set of 148 unique *L. pneumophila *strains with the statistical five marker model built with 109 unique strains

	Patient derived	Environmental	Total
Positive Test result	34	35	69

Negative Test result	0	79	79

Total	34	114	148

			

sensitivity	100%		

specificity	69%		

### Identity of selected markers

Sequence analysis of the 5 selected markers enabled comparison with known *Legionella *sequences. Three out of the 5 markers showed variable presence in the 4 completely sequenced clinical isolates of *L. pneumophila *(Table 4) whereas two other markers were present in all 4 sequenced genomes. Since these markers showed differential presence in the strain population we have analyzed, this indicated that the sequenced genomes do not completely cover genomic variation in *L. pneumophila*. One marker (11A2) represents part of *L. pneumophila *plasmid pLPP; it cannot be concluded based on the current data whether the plasmid is freely present in the bacterial cell or whether (part of) the plasmid DNA is inserted in the genome. The other 4 markers represent chromosomally derived fragments probably related to cell wall synthesis (19H4 lipopolysaccharide synthesis, 10B12 CMP-N-acetylneuraminic acid synthetase), conjugative coupling factor (24B1) and hypothetical (8D6). Details on these markers will be presented elsewhere.

**Table 4:** Sequence homologies of the 5 predictive markers when compared to available sequences for *L. pneumophila *strains Paris (lpp), Philadelphia (lpg), Lens (lpl), Corby (LPC) and *L. pneumophila *str. Paris plasmid (plpp)

Marker name	Homologues in available genome sequences
11A2	plpp0001

19H4	lpp0831; lpg0766; lpl0807; LPC_2526

10B12	lpp0816/0817; lpg0750/0751; lpl0787/10788; LPC_2541/2542

24B1	lpp2398/2399; LPC_1880/1881

8D6	lpg0514; lpl0552

Absence of homologues indicates that no high similarity homologue is present. The sequences are accessible through GenBank accession numbers HM584933-HM584937).

## Discussion

To study the genomic diversity within *L. pneumophila *in more detail a mixed-genome microarray was developed for this species. This approach has shown to be very useful for studying genome diversity in other bacterial species such as *Streptococcus pyogenes *and *Enterococcus faecium *[19,20]. By selecting clinical as well as environmental strains for microarray construction gene loss and acquisition in both groups of strains can be identified. This is a clear advantage over the more commonly used genome-sequencing approach which mostly focuses on clinical strains, thereby dismissing the ability to identify differences between environmental and clinical strains. Here we show the feasibility of this discrimination for *L. pneumophila*, the causative microorganism for Legionnaires' disease. Clinical isolates of this bacterial species could be identified in 100% of the cases based on a model consisting of 5 DNA markers. This result will enable a more detailed analysis of *Legionella*-contaminated water sources and help to recognize potential public health dangers before the onset of new LD outbreaks.

To achieve this result, two factors are of utmost importance: the use of an unbiased genomics approach and the availability of a well-defined and well-characterized bacterial strain collection. When studying pathogenicity, the availability of clinical strains is relatively straightforward, especially if a surveillance program is ongoing, as is the case in The Netherlands for *L. pneumophila*. Collecting reliable environmental strains, however, is far more difficult. Probably the biggest issue in considering a strain as non-pathogenic is the question of whether the specific isolate lacks the potential to be pathogenic or just did not have a chance to exhibit pathogenic behavior. In the case of the Dutch Legionella surveillance program, not only were patient isolates collected, but also attempts were made to track the infectious source by analyzing the environment in which a patient lived. This was done by analyzing hot water systems in homes and water systems in caravans used by patients, and analyzing swimming pools, saunas and other potential sources visited by patients shortly before getting ill. In a number of cases a match could be made between an environmental isolate and the patient isolate but, in most cases, isolates identified in a patient's environment were shown to be different from the patient strain. It seems very likely that the patient in these cases had been in contact with the specific environmental isolate and did not become ill, but did become ill from another isolate; this, in combination with the registration history of notified cases for those potential sources, leads us to conclude that this is the safest definition currently possible for non-pathogenic strains. Therefore, we have used this strain collection for a genomic analysis of differences between clinical and environmental strains, while understanding that it is still possible for an environmental strain to be pathogenic.

We have generated genomic fingerprints for 257 unique *L. pneumophila *strains and used these to select DNA markers enabling discrimination between clinical and environmental *L. pneumophila *strains. The rationale behind this approach is the hypothesis that bacterial species are not a homogeneous group of organisms but contain significant individual variation at the nucleotide level (single nucleotide polymorphisms) and at the genome composition level (one or more genes, operons, and so on). In order to analyze genome composition variation, we have developed our genomotyping platform. When applied to pathogenic micro-organisms this type of analysis might lead to more detailed discrimination between the real pathogenic strains within such a species and the non- (or far less) pathogenic strains. Since genomic differences between bacterial strains probably lead to physiological differences, better ability to discriminate between strains may lead to better understanding of the pathogenicity process itself and to more focused diagnosis, treatment or therapy. In many cases, the major hurdle for applying this approach is the lack of well-characterized bacterial strain collections. In the case of *L. pneumophila*, we were fortunate to have access to a very well-characterized strain collection with respect to clinical as well as non-clinical (environmental) isolates. The use of this strain collection combined with our genomotyping platform and novel way of analyzing genomic data has led to the selection of five DNA markers that together can discriminate between clinical and environmental *L. pneumophila *strains with high reliability. Genetic programming was instrumental in the selection of this minimal set of predictive markers and in the definition of the relationships between the individual markers. The inclusion of these relationships is an essential step since a univariate approach looking at each marker individually would never have selected some of our five markers. In addition, some markers are important because they are nearly always absent in clinical strains and would never have been identified if environmental strains had not been included during array construction. These aspects are often ignored when identifying predictive markers, but they fit perfectly with the kind of relationships that play a central role in biology.

Attempts have been made previously to identify pathogenicity markers by, *e.g.*, comparing the complete genome sequences of clinical and environmental strains. The disadvantage of such an approach is the fact that there are usually dozens to hundreds of differences detected, only a few of which may be important with regard to the property under study. Furthermore, in most cases there is an emphasis on the clinical strains (for example: four genomic sequences are publicly available for *L. pneumophila*, all for clinical strains [21,22]. Without the ability to compare these to environmental strains it will be very difficult to reach a better understanding of pathogenicity. To really start to understand pathogenicity a population-based approach is necessary. This kind of approach has been used for epidemiology research in microbiology, but the methods used in those cases (Pulsed Field Gel Electrophoresis, Restriction Fragment Length Polymorphism, AFLP) have only a limited resolution, or they have a high resolution but focus on genes not involved in pathogenicity (Multi Locus Sequence Typing (also known as Sequence Based Typing) of core genome/house-keeping genes [23,24]. Cazalet et al [7] described a multigenomic analysis of a large number of *Legionella *strains, but their aim was not to select a minimal set of predictive markers. Furthermore, their analysis was based on a microarray derived from genomic sequences of clinical strains only. In the work presented here, a combination of two different approaches was used, combining a population-based view with a detailed molecular analysis. When performed as described here, this is a technically feasible and relatively inexpensive way to identify predictive markers. There are multiple ways to translate these markers into practical applications which, in this case, can have a significant impact on control of LD. Dutch regulations are focused on finding legionellae in water systems, especially in relation to vulnerable populations in care facilities, but also in relation to hotels and public buildings. Positive cultures of water samples call for immediate action, including implementation of costly measures and potential closing of wards or buildings. The ability to differentiate between clinical and environmental strains can be used as a tool to fine-tune control procedures. Furthermore, in the case of *L. pneumophila *as well as other micro-organisms, the described technique may act as a starting point for the development of new diagnostics and therapeutics.

## Conclusions

In conclusion, a novel way of studying the genome composition of *L. pneumophila *combined with the availability of a *L. pneumophila *strain collection derived from a surveillance program allowed us to search for genomic differences between patient-derived and environmental strains of this bacterium. By using genetic programming 5 markers could be selected which together enable a reliable identification of clinical strains. This opens up the way for novel diagnostic approaches better suited to recognize clinical *L. pneumophila *strains. This may also help to improve the confounding situation that despite increased awareness and preventive measures the incidence of LD is still increasing in The Netherlands. Furthermore, detailed analysis of the identified markers may lead to better understanding of *L. pneumophila *virulence and to improved protective measures.

## Methods

### Strains

All strains used in the research described here are derived from the National Outbreak Detection Program [25]. Detailed information on the strains can be found in additional file 1.. In this Table the strains are divided into three groups: the strains used to construct the genomic library and the microarray, the strains used to develop the predictive model (also referred to as training set) and the strains used to test the model (also referred to as test set).

### Genomic analysis

Comparative genome hybridization experiments were performed with a mixed-genome *L. pneumophila *microarray. For the construction of this microarray, eight *L. pneumophila *strains were selected based on their diversity (serotype and origin, see additional file 1). Genomic DNA of these strains was isolated, mixed in equimolar amounts, sheared to approximately 1.5 kb fragments and used for construction of a genomic library in pSMART (Lucigen), as earlier described [19,20]. A total of 3,360 recombinant clones were collected; inserts were amplified and spotted on aldehyde-coated glass slides, as described previously [19,20]. Sequencing of amplicons of randomly selected clones confirmed the presence of *L. pneumophila *specific fragments.

This microarray (which theoretically had more than full genome coverage) was used to analyze the genome composition of the collection of 257 unique *Legionella *strains by comparing labeled DNA from each strain with a reference containing labeled DNA from the mixture of strains used for array construction (see below for labeling details). This leads to a fingerprint encompassing over 3,000 different markers for each strain. The total number of experiments performed for the training and test sets were 133 and 213 respectively (adding up to 346 in total), but since this includes replicates (both technical and biological) most calculations are based on a selection of unique strains only. In total 257 unique strains have been used for the work described here: 109 strains in the training set and 148 in the test set. In Table S1 all 133 and 213 experiments are given together with strain names and replicate indications. Experiments for which the same strain name is given multiple times represent technical replicates, whereas experiments for which strain names are different but the same replicate number is shown represent biological replicates (with the exception of replicate number 0 which indicates unique data sets). Reproducibility of the analysis was tested by running multiple repeat experiments (duplicate or triplicate analyses of identical strains).

The genomic DNA of specific *L. pneumophila *strains was labeled fluorescently with Cy5-dUTP using the BioPrime system (Invitrogen). Reference DNA (0.5 μg from the mix used for microarray construction) was labeled with Cy3-dUTP. Both sets of samples were hybridized on pre-hybridized microarrays overnight at 42°C. For scanning, a ScanArray TM Express (Packard BioScience) was used. Quantification of hybridization signals was done using ImaGene version 5.6 software (BioDiscovery).. To correct for differences in labeling, hybridization conditions, slide quality, and scanning circumstances, each slide was normalized independently. At first, ratios of Cy5 minus background to Cy3 minus background were calculated. Filtering was applied to exclude spots with flags; for estimating the correction factor in normalization, only spots were included with Cy3 values larger than two times background. Mean ratios were then calculated and applied to each independent ratio resulting in normalized ratios for each spot. After obtaining full array data for 133 isolates, a selection of markers showing variation between individual strains was made. In total 480 markers were selected, 18 of which represented constant (always present) markers which were needed to normalise data between microarrays. The other 462 markers all represent markers showing clear variation in presence between individual strains. The number of 480 markers was chosen for practical reasons since this number fitted within the microarray format used for the further experiments which was a multi-well microarray, allowing for higher throughput. All data for the test set strains were obtained from those multi-well microarrays. All protocols for labeling, hybridization and quantification remained unchanged. Normalisation between microarrays was now based on the results obtained for the 18 constant markers by calculating mean ratios based on these markers only and applying the normalisation factor then to each independent ratio resulting in normalized ratios for each spot. Normalised raw data for both the training (133 dataset) and test (213 dataset) sets are given in additional files 2 and 3.

Hierarchical clustering of differentiating biomarkers from all strains was done with TIGR software (available at http://www.tigr.org/software/tm4, [26]), using average linkage and Pearson correlation as distance matrix.

### Conversion of data to binary values

A closer look at the data obtained for the variable markers showed that the binary nature of the data distribution over the strain population was evident. We developed a novel approach for binarization which works as follows. First, we scaled our data symmetrically around 1 using a calculation (if R > 1 then 2-1/R) in which R is the ratio of specific strain signal divided by the reference pool signal (we introduced R_lim _for this which was calculated with the Excel formula R_lim _= if(R < 1, R, 2-1/R). In this way all ratios are in the 0-2 range centered around 1 in which low values are nearing 0 and high values are nearing 2. Then, we ordered all data for each spot in an increasing row and plotted these values (see additional file 4 for examples). We performed this separately for the training set (133 values) and the test set (213 values). Next, we empirically determined the cut-off for each spot from these plots and classified all individual datapoints as being absent (0) or present (1), based on this cut-off. In cases where no clear cut-off could be determined, all datapoints were classified as being present (1). In cases where multiple cut-offs were possible, we decided to apply all cut-offs by splitting up these markers and using all variants for further data analysis. An example of such a situation is shown in additional file 4, panel D, which a data distribution which clearly suggests a triple distribution of data. It is tempting to speculate that such a distribution could represent a marker which is either absent, present in a single copy or present in multiple copies in a specific *L. pneumophila *strain. One of the markers in the five marker model (11A2-b) in fact shows this distribution and this marker appears to represent part of a plasmid. Attempts were made to automate the binarisation process: this worked out for a single cut-off but not for multiple cut-offs. Binarized data for both the training (133 dataset) and test (213 dataset) sets are given in additional files 5 and 6.

### Selection of discriminating markers

Genetic programming analysis was performed with 133 data sets. Careful examination of all the data showed that this set of 133 contained 109 unique strains. The redundancy was caused mainly by the inclusion of replicate experiments (both technically and biologically). The same was true for the reserved test set which originally contained 213 data sets; this decreased to 160 when removing replicates and to 148 when data sets overlapping the training set were also removed. Additional file 7 shows that the inclusion of these replicate data sets did not significantly influence the stability or predictive value of the model. **A **shows the outcome when all individual data sets were considered unique, **B **shows the outcome when replicate experiments were removed and **C **shows the outcome when data sets overlapping between the training and test sets were removed from the test set.

## Abbreviations

LD: Legionnaire's disease; CGH: Comparative Genome Hybridization

## Authors' contributions

EY, JdB, WvdM, RM and FS designed the project. EY and JdB collected all bacterial strains and collected data, MC, RM and FS developed the microarray, collected all microarray data and performed data preprocessing and processing. AA and BW identified predictive markers by performing Genetic programming analysis on microarray data. FS and EY wrote the paper. All authors discussed the results and commented on the manuscript.

All authors have read and approved the final manuscript

## Supplementary Material

Additional file 1 **SupplTable1 List of strains**. list of strains used in this work. Click here for file

Additional file 2 **supplTable2 rawdatatrainingset**. non-binarized microarray data used as a trainingset for constructing the predictive model. Click here for file

Additional file 3 **supplTable3 rawdatatestset**. non-binarized microarray data used as a testset for validating the predictive model. Click here for file

Additional file 4 **Supplementary Figure 1 binarisation examples**. Figure showing examples of the binarization process used for the microarray data. Click here for file

Additional file 5 **supplTable4 binarizeddatatrainingset**. binarized microarray data used as a trainingset for constructing the predictive model. Click here for file

Additional file 6 **supplTable5 binarizeddatatestset**. binarized microarray data used as a testset for validating the predictive model. Click here for file

Additional file 7 **supplementary table 6**. performance of the predictive model with different sets of data used. Click here for file
