# Supplementary material for: Comparative genome analysis of a large Dutch Legionella pneumophila strain collection identifies five markers highly correlated with clinical strains
Source: BMC Genomics. 2010 Jul 15;11:433. doi: 10.1186/1471-2164-11-433 (PMC3091632; doi:10.1186/1471-2164-11-433)
Supplement: Additional file 4 — Supplementary Figure 1 binarisation examples. Figure showing examples of the binarization process used for the microarray data. [file 1471-2164-11-433-S4.DOC]

A

B

C

D

**Figure S1** Four examples of representative ratio distributions of single datapoints over all experiments. **A** represents a situation in which no cut-off could be assigned and represents a constant marker present in all strains. **B** represents a situation in which the marker is absent in most strains and present only in some strains whereas **C** represents a more equal distribution in the number of cases in which this marker is present and absent. In both **B** and **C** cut-off assignment is relatively straightforward. **D** represents a situation in which multiple cut-offs can be assigned.
