# Supplementary material for: Comparative genome analysis of a large Dutch Legionella pneumophila strain collection identifies five markers highly correlated with clinical strains
Source: BMC Genomics. 2010 Jul 15;11:433. doi: 10.1186/1471-2164-11-433 (PMC3091632; doi:10.1186/1471-2164-11-433)
Supplement: Additional file 7 — supplementary table 6. performance of the predictive model with different sets of data used. [file 1471-2164-11-433-S7.DOC]

| **A** |  |  |  |
| --- | --- | --- | --- |
| **Training set** |  |  |  |
|  | Patient derived | Environmental | Total |
| Positive Test result | 66 | 23 | 89 |
| Negative Test result | 0 | 44 | 44 |
| Total | 66 | 67 | 133 |
| sensitivity | 100% |  |  |
| specificity | 66% |  |  |
|  |  |  |  |
| **Test set** |  |  |  |
|  | Patient derived | Environmental | Total |
| Positive Test result | 56 | 50 | 106 |
| Negative Test result | 0 | 107 | 107 |
| Total | 56 | 157 | 213 |
| sensitivity | 100% |  |  |
| specificity | 68% |  |  |
|  |  |  |  |
| **B** |  |  |  |
|  |  |  |  |
| **Training set** |  |  |  |
|  | Patient derived | Environmental | Total |
| Positive Test result | 49 | 23 | 72 |
| Negative Test result | 0 | 37 | 37 |
| Total | 49 | 60 | 109 |
| sensitivity | 100% |  |  |
| specificity | 62% |  |  |
|  |  |  |  |
| **Test set** |  |  |  |
|  | Patient derived | Environmental | Total |
| Positive Test result | 43 | 35 | 78 |
| Negative Test result | 0 | 82 | 82 |
| Total | 43 | 117 | 160 |
| sensitivity | 100% |  |  |
| specificity | 70% |  |  |
|  |  |  |  |
| **C** |  |  |  |
|  |  |  |  |
| **Training set** |  |  |  |
|  | Patient derived | Environmental | Total |
| Positive Test result | 49 | 23 | 72 |
| Negative Test result | 0 | 37 | 37 |
| Total | 49 | 60 | 109 |
| sensitivity | 100% |  |  |
| specificity | 62% |  |  |
|  |  |  |  |
| **Test set** |  |  |  |
|  | Patient derived | Environmental | Total |
| Positive Test result | 34 | 35 | 69 |
| Negative Test result | 0 | 79 | 79 |
| Total | 34 | 114 | 148 |
| sensitivity | 100% |  |  |
| specificity | 69% |  |  |
